# From barcode entropy to metric entropy

**DOI:** 10.1007/s11784-026-01301-y

**Published:** 2026-05-26

**Authors:** Erman Çineli, Viktor L. Ginzburg, Başak Z. Gürel

**Affiliations:** 1 ETH Zürich, Rämistrasse 101, 8092 Zurich, Switzerland; 2 Department of Mathematics, UC Santa Cruz, Santa Cruz, CA 95064 USA; 3 Department of Mathematics, University of Central Florida, Orlando, FL 32816 USA

**Keywords:** Topological entropy, metric entropy, Lagrangian and Legendrian submanifolds, periodic orbits, Floer homology, symplectic homology, persistence modules, 53D40, 37C40, 37J12, 37J46, 37J55

## Abstract

We establish a connection between barcode entropy and metric entropy. Namely, we show that the barcode entropy bounds the metric entropy from below for a measure from a specific class of invariant measures associated with a pair of Lagrangian or Legendrian submanifolds, which we call *pseudo-chord measures*. This inequality refines, via the variational principle, the previously known upper bound of barcode entropy by topological entropy.

## Introduction

In this paper, we continue the study of barcode entropy and establish its connection with metric entropy for a certain class of invariant measures of Hamiltonian diffeomorphisms and Reeb flows.

Barcode entropy is a Floer theoretic counterpart of topological entropy. For Hamiltonian diffeomorphisms, the barcode entropy is the exponential growth rate of the number of not-so-short bars in the filtered Floer homology as a function of iteration. For Reeb flows, one looks at this number for the filtered symplectic homology as a function of the action. These invariants come in two forms: absolute and relative. The absolute version uses fixed point Floer homology or symplectic homology and thus provides a lower bound on the growth of periodic orbits. In the relative version, we have a pair of Lagrangian (or Legendrian) submanifolds fixed and work with filtered Lagrangian Floer homology or wrapped Floer homology. Thus, the relative barcode entropy gives a lower bound on the exponential growth rate of the number of chords. The latter lower bounds are more robust under small perturbations of submanifolds than the growth of the actual number of chords. This fact leads to a relation between barcode entropy and the volume growth and ultimately topological entropy.

Barcode entropy was originally introduced in [10] in the Hamiltonian setting and then extensively studied in [9, 11, 14, 16–18, 20, 34]. We will review the definitions and basic properties in Sect. 2.1.

The feature of barcode entropy which is absolutely essential for what follows is that it bounds topological entropy from below. (In some cases, there is also an opposite inequality capturing topological entropy of hyperbolic sets, but this fact is less important for our purposes in this paper.) Here, we refine this inequality by showing that in the relative case the barcode entropy bounds from below the metric entropy for a measure from a specific class of invariant measures associated with a pair of Lagrangian or Legendrian submanifolds. We refer to this class as the *pseudo-chord measures* and it is essential that pseudo-chord measures depend on the Lagrangian/Legendrian submanifolds. Then the original bound with topological entropy simply follows from the fact that the metric entropy is bounded from above by the topological entropy; see, e.g., [25]. The main new and non-obvious point here is the definition of a pseudo-chord measure given in Sects. 2.2 and 2.4.

A similar inequality holds for absolute barcode entropy with chord measures replaced by what we call *pseudo-periodic measures*; see Sect. 2.3. However, this class of measures is quite large and includes all ergodic measures. As a consequence, the inequality, taken literally, does not give new information; for it follows from the variational principle and the known relations between barcode entropy and topological entropy [10, 16].

## Main results

### Barcode entropy

In this section, we briefly review the definition of barcode entropy. We start with the case of Hamiltonian diffeomorphisms and then recast the definitions in the framework of Reeb flows. For further details and references, we refer the reader to [10, 14].

Let $L_0$ and *L* be two closed, monotone, Hamiltonian isotopic Lagrangian submanifolds in a symplectic manifold *M*. We require *M* to be compact or sufficiently well-behaved at infinity (e.g., convex) so that the filtered Floer homology for the pair $(L_0,L)$ is defined. This homology is not a persistence module in the conventional sense, but its barcode is still defined at least when $L_0$ and *L* are transverse and each bar is taken up to a shift; see [42]. Denote by ${\mathfrak b}_\epsilon (L_0,L)$ the number of bars of length greater than ϵ>0. This definition extends to not necessarily transverse pairs essentially by continuity [10, Sec. 4].

Next, let $\varphi :M\rightarrow M$ be a compactly supported $C^\infty$-smooth Hamiltonian diffeomorphism. By definition, the ϵ*-barcode entropy of* φ *relative to* $L_0$ *and* *L* is

2.1 $$\begin{aligned} {\hbar }_\epsilon (\varphi ; L_0,L):=\limsup _{k\rightarrow \infty }\frac{\log ^+ b_\epsilon (L_k,L)}{k}\text { where } L_k:=\varphi ^k(L_0). \end{aligned}$$

Here and throughout the paper, the logarithm is taken with the same base as in the definition of topological entropy, and $\log ^+=\log$ except that $\log ^+0=0$. Then the *barcode entropy of* φ *relative to* $L_0$ *and* *L* is

2.2 $$\begin{aligned} {\hbar }(\varphi ;L_0,L):=\lim _{\epsilon \rightarrow 0+} {\hbar }_\epsilon (\varphi ; L_0,L)\in [0, \infty ]. \end{aligned}$$

When *M* is closed and monotone or weakly monotone, we can apply this construction to the Hamiltonian diffeomorphism ${ i d}\times \varphi$ of the symplectic square M×M with $L_0=L$ taken to be the diagonal $\Delta \subset M\times M$. As a result, we obtain the (absolute) ϵ-barcode entropy $\hbar _\epsilon (\varphi )$ of φ and the *(absolute) barcode entropy* $\hbar (\varphi )$. More explicitly, $\hbar _\epsilon (\varphi )$ is the exponential growth rate, (2.1), of the number, ${\mathfrak b}_\epsilon (\varphi ^k)$, of bars of length greater than ϵ>0 in the filtered Floer homology of $\varphi ^k$.

Barcode entropy is closely related to topological entropy. Namely, [10, Thm. A] asserts that

2.3 $$\begin{aligned} \hbar (\varphi ; L_0,L)\le \operatorname {h}_{\operatorname {top}}(\varphi ) \end{aligned}$$

and, in particular,

2.4 $$\begin{aligned} \hbar (\varphi )\le \operatorname {h}_{\operatorname {top}}(\varphi ). \end{aligned}$$

As a consequence, barcode entropy is finite. (The requirement that φ is $C^\infty$-smooth is essential at least on the level of proofs; for the argument relies on Yomdin’s theorem [43].) Inequality (2.4) can be strict: there exists a Hamiltonian diffeomorphism φ of a closed symplectically aspherical manifold such that $\hbar (\varphi )=0$ but $\operatorname {h}_{\operatorname {top}}(\varphi )>0$; see [9].

Furthermore, when K⊂M is a compact hyperbolic invariant set of φ,

2.5 $$\begin{aligned} \operatorname {h}_{\operatorname {top}}(\varphi |_K)\le \hbar (\varphi ) \end{aligned}$$

by [10, Thm. B]. Therefore, combining these inequalities with the results of Katok from [24], we see that

$$\begin{aligned} \hbar (\varphi )=\operatorname {h}_{\operatorname {top}}(\varphi )\text { when } \dim M=2; \end{aligned}$$

[10, Thm. C]. Inequality (2.5) is extended to the relative case in [34, Thm. 1]. Namely, assume that $L_0$ and *L* intersect *K* and that $L_0$ contains a ball centered at $p\in L_0\cap K$ in the unstable manifold of *K* at *p* and *L* contains a ball centered at $q\in L\cap K$ in the stable manifold. Furthermore, we require *K* to be locally maximal and topologically transitive. Then

2.6 $$\begin{aligned} \operatorname {h}_{\operatorname {top}}(\varphi |_K)\le \hbar (\varphi ;L_0,L). \end{aligned}$$

Formally speaking, this inequality does not directly imply (2.5), i.e., the two inequalities are logically independent.

These constructions and results extend to Reeb flows. Consider the Reeb flow $\varphi :=\{\varphi ^t\mid t\in {\mathbb R}\}$ on the boundary *M* of a Liouville domain *W*. Then the filtered symplectic homology of *W* is a genuine persistence module (see, e.g., [14, 16]), and we denote by ${\mathfrak b}_\epsilon (s;\varphi )$ the number of bars of length greater than ϵ>0 beginning below $s\in {\mathbb R}$. (Here, the grading of the homology is inessential and we need not make any assumptions on $c_1(TW)$.) Then the ϵ-barcode entropy and the barcode entropy of φ is defined similarly to (2.1) and (2.2):

2.7 $$\begin{aligned} {\hbar }_\epsilon (\varphi ):=\limsup _{s\rightarrow \infty }\frac{\log ^+ {\mathfrak b}_\epsilon (s;\varphi )}{s}\text { and } {\hbar }(\varphi ):=\lim _{\epsilon \rightarrow 0+} {\hbar }_\epsilon (\varphi ). \end{aligned}$$

In the relative version, following [17], we start with closed Legendrian submanifolds $L_0$ and *L* in M=∂W and require $L_0$ and *L* to admit exact compact Lagrangian fillings $\hat{L}_0$ and $\hat{L}$ to *W* such that $L_0=\partial \hat{L}_0$ and $L=\partial \hat{L}$. Then we can also make $\hat{L}_0$ and $\hat{L}$ be asymptotically conical Lagrangian submanifolds in *W*. The latter condition means that, say, $\hat{L}$ has the form $\hat{L}=L\times (1-\epsilon ,1]$ within a symplectic collar $M\times (1-\epsilon , 1]$ of M=∂W in *W*.

Then in place of symplectic homology we use the wrapped Floer homology persistence module for $(\varphi , \hat{L}_0,\hat{L})$ to define ${\mathfrak b}_\epsilon (s; L_0,L)$, the number of bars of length greater than ϵ beginning below *s*, and the barcode entropy ${\hbar }(\varphi ; L_0, L)$ is defined similarly to (2.7). (We have suppressed φ in the notation for the sake of brevity.) In fact, ${\mathfrak b}_\epsilon (s; L_0,L)$ is independent of the choice of $\hat{L}_0$ and $\hat{L}$ up to an additive constant, and hence ${\hbar }(\varphi ; L_0, L)$ is completely determined by φ and $L_0$ and *L*. Only the existence of exact Lagrangian fillings $\hat{L}_0$ and $\hat{L}$ matters.

With these definitions, the above results also hold for Reeb flows. The Reeb analogue of (2.3) is proved in [17]. The absolute version of this inequality, (2.4), which now does not directly follow from its relative counterpart is established in [16]; see also [14]. The Reeb counterpart of the lower bound, (2.5), is [14, Thm. B], and its relative version, (2.6), is the main result of [18]. Finally, we again have

$$\begin{aligned} \hbar (\varphi )=\operatorname {h}_{\operatorname {top}}(\varphi )\text { when } \dim M=3 \end{aligned}$$

using [28, 29]; see [10]. For geodesic flows, these results, other than (2.6), are directly proved in [20] in the context of Morse theory.

#### Remark 2.1

(Applications) The lower bounds between barcode entropy and topological entropy, relative and absolute, for Reeb and geodesic flows along the lines of (2.3) and (2.4) were established in [14, 16, 17, 20]. They encompass several “classical” results relating positivity of topological entropy to topology of the underlying Riemannian manifold or symplectic topology of the contact manifold. The first of these is Dinaburg’s theorem, [15], giving a lower bound for the topological entropy of a geodesic flow via the growth of the fundamental group. Likewise, the lower bound from [36] for the topological entropy by the exponential growth rate of the homology of the based loop space immediately follows from [20]. Both of these results have been generalized to Reeb flows on unit (co)tangent bundles in [30] and [19, Prop. 1.8], and this generalization is in turn covered by [17]. Several yet more general lower bounds on topological entropy of Reeb flows via the growth of various types of symplectic or contact homology have been proved in [3–6, 33]. Some of these lower bounds also follow, directly or at least conceptually, from the results of [14, 16, 17].

#### Remark 2.2

Connections between the properties of Floer homology type persistence modules and symplectic dynamics go far beyond topological and metric entropy. We refer the reader to, e.g., [8, 12, 13, 21, 22, 27, 37, 38, 40–42] for an admittedly incomplete list of sample results and further discussion and references.

### Lagrangian pseudo-chord measures

The definition of approximate chord measures and pseudo-chord measures, which are central to our results, can be given in a very general context of topological dynamics.

Let $\varphi :M\rightarrow M$ be a compactly supported diffeomorphism of a smooth manifold *M*. By definition, the *k*-orbit $\mathcal {O}:=\mathcal {O}_k(x)$ of φ through x∈M is a sequence $\{x, \varphi (x), \ldots , \varphi ^{k}(x)\}$. To $\mathcal {O}$, we associate the probability measure

2.8 $$\begin{aligned} \delta _\mathcal {O}=\frac{1}{k+1}\sum _{i=0}^k \delta _{\varphi ^i(x)}. \end{aligned}$$

Likewise, we associate the probability measure

2.9 $$\begin{aligned} \delta _\Gamma =\frac{1}{m}\sum _{i=1}^m \delta _{\mathcal {O}_k(x_i)} \end{aligned}$$

to a collection of *k*-orbits $\Gamma =\{\mathcal {O}_k(x_1),\ldots ,\mathcal {O}_k(x_m)\}$. The points $x_i\in M$ are not required to be distinct, and thus Γ is a multiset. Note, however, that here the “length” *k* is the same for all orbits in the collection.

Next, let $L_0$ and *L* be two subsets *M*. The case we are actually interested in is where $L_0$ and *L* are properly embedded submanifolds of *M*. Fix an open set U⊃L. The reader should think of *U* as a small tubular neighborhood of *L* when the latter is a submanifold. A *U-approximate* *k-chord* (or just an approximate chord when *U* and *k* are clear from the context) is a *k*-orbit $\mathcal {O}_k(x)$ with $x\in L_0$ and $\varphi ^k(x)\in U$.

Assume that for a sequence $k_j\rightarrow \infty$ and a sequence $\Gamma _j=\{\mathcal {O}_{k_j}(x_{j1}),\ldots , \mathcal {O}_{k_j}(x_{{jm_j}})\}$ of collections of *U*-approximate $k_j$-chords the measures $\delta _{\Gamma _j}$ converge to a measure μ in the weak* topology. Then we say that μ is a *U-approximate chord measure* (associated with $L_0$ and *L*). Note that μ is automatically an invariant probability measure. The set of *U*-approximate chord measures is weak*-closed. This follows by the diagonal process from the fact that the set (with the weak* topology) of probability measures on a compact metric space is metrizable [39].

Furthermore, an invariant probability measure μ is said to be a *pseudo-chord measure* (again, associated with $L_0$ and *L*) if $\mu =\lim \mu _\ell$ for a sequence $U_\ell$, $\ell \in {\mathbb N}$, of open neighborhoods of *L* shrinking to *L* and a sequence $\mu _\ell$ of $U_\ell$-approximate chord measures. Here, we say that $U_\ell$ is shrinking to *L* if

$$\begin{aligned} \bigcap _\ell U_\ell =L \end{aligned}$$

and the neighborhoods $U_\ell$ are nested, i.e., $U_1\supset U_2\supset \cdots \supset L$.

More explicitly, μ is a pseudo-chord measure if there exists a sequence of neighborhood $U_\ell$ shrinking to *L* and a sequence of collections $\Gamma _\ell$ of $U_\ell$-approximate $k_\ell$-chords with $k_\ell \rightarrow \infty$ such that $\delta _{\Gamma _\ell }\rightarrow \mu$. Note that here the chords comprising $\Gamma _\ell$ are not necessarily true chords from $L_0$ to *L* but only approximate chords. (Hence, “pseudo” in the term.) In general, μ cannot be obtained as the limit of measures $\delta _\Gamma$ where Γ comprises genuine chords from $L_0$ to *L*; see Remark 2.6. We will call such measures *genuine (or true) chord measures*.

In these definitions, we could have assumed that *M* is just a metric space and φ is a continuous map. However, in such a great generality the definition is probably meaningless. On the other hand, as we have already mentioned, the case where $L_0$ and *L* are properly embedded submanifolds of *M* is already of interest; see Sect. 3.1.

In this paper, we are concerned with the role of pseudo-chord measures in symplectic dynamics. Thus, we will require $L_0$ and *L* to be closed Lagrangian submanifolds of a symplectic manifold *M* and $\varphi :=\varphi _H:M\rightarrow M$ to be a compactly supported Hamiltonian diffeomorphism. In this setting, we say that a pseudo-chord measure associated with $L_0$ and *L* is *Lagrangian*. For the sake of simplicity, we will assume that $L_0$ and *L* are monotone, although this condition can be significantly relaxed. As in the previous section, the manifold *M* is required to be closed or sufficiently nice at infinity (e.g., convex or tame) so that Lagrangian Floer homology for closed monotone Lagrangian submanifolds is defined.

The sets of approximate chord measures or pseudo-chord measures can be empty. We say that *L* is *accessible* from $L_0$ if for every neighborhood *U* of *L* we have $L_k\cap U\ne \emptyset$ for infinitely many $k\in {\mathbb N}$, where $L_k = \varphi ^k(L_0)$. This is equivalent to that the set of pseudo-chord measures associated with $L_0$ and *L* is non-empty. Clearly, *L* is accessible from $L_0$ whenever $\hbar (\varphi ; L_0,L)>0$.

#### Theorem 2.3

Let $L_0$, *L*, *U* and φ be as above. Assume that *L* is accessible from $L_0$. Then there exists a *U*-approximate chord measure μ associated with $L_0$ and *L* such that

2.10 $$\begin{aligned} \hbar (\varphi ; L_0,L)\le \textrm{h}_\mu (\varphi ). \end{aligned}$$

Shrinking *U* to *L*, passing to the limit and using the fact that the map $\mu \mapsto \textrm{h}_\mu$ is upper semi-continuous in μ (see [35]) we obtain the following. Recall that $\operatorname {supp}(\varphi )$ is compact, and hence the set of probability measures on $\operatorname {supp}(\varphi )$ is compact in the weak* topology.

#### Corollary 2.4

There exists a pseudo-chord measure μ associated with $L_0$ and *L* satisfying (2.10).

Since $\textrm{h}_\mu (\varphi )\le \operatorname {h}_{\operatorname {top}}(\varphi )$ for any probability measure μ, as a consequence of Corollary 2.4 we recover the lower bound, (2.3), on the topological entropy via the barcode entropy from [10, Thm. 5.1]: $\hbar (\varphi , L_0,L)\le \operatorname {h}_{\operatorname {top}}(\varphi )$. Also note that the accessibility condition plays a purely formal role in these results: without it the left-hand side is zero and the right-hand side is not defined. It is essential for the proof of Theorem 2.3 that the chords in question are only approximate rather than genuine chords from $L_0$ to *L*, i.e., that the end-points belong to *U* but not necessarily to *L*. We do not know if Corollary 2.4 would still hold with the set of pseudo-chord measures replaced by the more narrow class of genuine chord measures; cf. Remark 2.6.

The proof of Theorem 2.3, given in Sect. 3.1, comprises two steps and utilizes yet another entropy-type invariant—the volume growth entropy $\operatorname {h}_{\operatorname {vol}}(\varphi ; L_0,U)$ defined in that section. The first step is the inequality $\hbar (\varphi ; L_0, L)\le \operatorname {h}_{\operatorname {vol}}(\varphi ; L_0,U)$ established in [10]. The second step is the inequality $\operatorname {h}_{\operatorname {vol}}(\varphi ; L_0,U)\le \textrm{h}_\mu (\varphi )$ which holds in much greater generality and is based on (the proof of) Yomdin’s theorem [43].

#### Remark 2.5

The class of all Lagrangian pseudo-chord measures, where we allow $L_0$ and *L* to vary, is quite large. For instance, it includes all ergodic measures; see Proposition 3.4. As a consequence, the variational principle holds for the class of all pseudo-chord measures, i.e., $\sup _\mu \textrm{h}_\mu (\varphi )=\operatorname {h}_{\operatorname {top}}(\varphi )$ where the supremum is taken over all such measures μ. At the same time it is not clear if a linear combination of pseudo-chord measures is again a pseudo-chord measure even with $L_0$ and *L* fixed. (The problem is that two such measures can have different time sequences.)

### Approximate periodic measures

Theorem 2.3 and the constructions from the previous section readily translate to a relation between absolute barcode entropy and metric entropy for measures associated with approximate periodic orbits. However, it turns out that the resulting class of measures is quite large and includes all ergodic measures similarly to the class of all pseudo-chord measures; see Remark 2.5. As a consequence, an analogue of (2.10) in this setting follows immediately from [10, Thm. 5.1] and the fact that $C^\infty$-maps have ergodic measures with maximal entropy, see [35]. Hence, formally speaking, the inequality does not give new information in this case. However, we find the construction illuminating enough to warrant a brief discussion.

Assume for the sake of simplicity that *M* is a closed weakly monotone symplectic manifold and $\varphi :M\rightarrow M$ is a Hamiltonian diffeomorphism. Let us apply the construction from Sect. 2.2 to the diagonal $\Delta =L_0=L$ in the symplectic product M×M and the Hamiltonian diffeomorphism ${ i d}\times \varphi$ of M×M. Pushing forward (approximate) chord measures on M×M to the second factor results in the notions of an approximate periodic measure and a pseudo-periodic measure. More explicitly, the construction is as follows.

Fix η>0 and a distance function *d* on *M*, and let

2.11 $$\begin{aligned} U_\eta :=\{(x,y)\in M\times M\mid d(x,y)<\eta \}. \end{aligned}$$

In the notation from Sect. 2.2, an η*-approximate* *k-periodic orbit* is an orbit $\mathcal {O}_k(x)$ such that $d\big (x, \varphi ^k(x)\big )<\eta$, i.e., $(x,\varphi ^k(x))\in U_\eta$. To a collection Γ of η-approximate *k*-periodic orbits (with the same *k*), we associate a probability measure $\delta _\Gamma$ given by (2.8) and (2.9). An η*-approximate periodic measure* μ is the weak* limit of a sequence of such measures $\delta _{\Gamma _j}$ as $k_j\rightarrow \infty$. Clearly, μ is automatically an invariant probability measure, and we could have replaced $U_\eta$ by an arbitrary neighborhood *U* of the diagonal. Furthermore, an invariant probability measure μ is said to be a *pseudo-periodic measure* if $\mu =\lim \mu _\ell$ for a sequence $\eta _\ell \rightarrow 0+$ and a sequence of $\eta _\ell$-approximate periodic measures $\mu _\ell$. In other words, μ is a pseudo-periodic measure if there exists a sequence $\eta _\ell \rightarrow 0+$ and a sequence $\Gamma _\ell$ of collections of $\eta _\ell$-approximate $k_\ell$-periodic orbits with $k_\ell \rightarrow \infty$ such that $\delta _{\Gamma _\ell }\rightarrow \mu$.

In this setting, applying Theorem 2.3 or Corollary 2.4 to the diagonal $L_0=L$ in M×M, replacing φ by ${ i d}\times \varphi$ and then projecting the measure to the second factor, we obtain the inequality

2.12 $$\begin{aligned} \hbar (\varphi )\le \textrm{h}_\mu (\varphi ) \end{aligned}$$

from Corollary 2.4. Here, we use the identity $\textrm{h}_\eta ({ i d}\otimes \varphi ) = \textrm{h}_{\pi _*\eta } (\varphi )$ which holds for any invariant probably measure η. (This can be seen using the Abramov–Rokhlin formula [1] and the fact that the fiberwise entropy is zero.) However, it is easy to show that in general every ergodic measure is pseudo-periodic; see Proposition 3.4. Hence, as we have already pointed out, (2.12) also readily follows from [10, Thm. 5.1] and the fact that the function $\mu \mapsto \textrm{h}_\mu (\varphi )$ is upper semi-continuous for $C^\infty$-diffeomorphisms, and hence φ admits ergodic measures with maximal entropy, i.e., $\textrm{h}_\mu (\varphi )=\operatorname {h}_{\operatorname {top}}(\varphi )$; [35].

#### Remark 2.6

(Pseudo-periodic vs. periodic). A *periodic measure* is defined as the limit of measures $\delta _\Gamma$ where all orbits comprising Γ are periodic, but possibly have different periods; see, e.g., [31]. On the other hand, when we apply the diagonal construction to genuine chord measures, we obtain the limits of measures $\delta _{\Gamma _\ell }$ where all orbits in $\Gamma _\ell$ have the same period. By analogy with the chord case, we would call such measures *genuinely periodic*. However, for discrete time the two notions agree as one can see from the proof of Proposition 3.5.

Furthermore, a periodic measure is pseudo-periodic in both continuous and discrete cases; see Proposition 3.5. The converse is not true: there are pseudo-periodic measures which are not periodic. For instance, let $\varphi :S^2\rightarrow S^2$ be an Anosov–Katok pseudo-rotation; [7]. This is an area-preserving diffeomorphism of $S^2$ with exactly three ergodic measures: two fixed points and the area form ω. Then ω is pseudo-periodic by Proposition 3.4. On the other hand, a periodic measure is necessarily a convex linear combination of the δ-measures at the fixed points. In particular, a pseudo-chord measure from $L_0$ to *L* need not be genuine chord measure, i.e., the limit of measures associated with true chords. Similar examples exist for broad classes of Reeb flows and Hamiltonian diffeomorphisms in all dimensions [23, 26].

We do not know exactly how large the class of pseudo-periodic measures is. While genuine periodic and ergodic measures are pseudo-periodic, it is not clear if a convex linear combination of pseudo-periodic measures is pseudo-periodic; cf. Remark 2.5.

### Reeb flows

The definitions and results from the previous two sections extend to Reeb flows in a straightforward way and here we only briefly comment on them.

Let $\varphi ^t$ be a flow on a closed manifold *M*. To an integral curve $\mathcal {O}:=\mathcal {O}_T(x):[0,T]\rightarrow M$, $t\mapsto \varphi ^t(x)$, we associate the measure $\delta _\mathcal {O}$ defined by

$$\begin{aligned} \delta _\mathcal {O}(f):=\frac{1}{T}\int _0^T f\big (\varphi ^t(x)\big )\,{\text {d}}t, \end{aligned}$$

where we view measures as elements of the dual space to $C^0(M)$. For a collection Γ of *m* not necessarily distinct orbits $\mathcal {O}_i:=\mathcal {O}_T(x_i)$, we set

$$\begin{aligned} \delta _\Gamma :=\frac{1}{m}\sum \delta _{\mathcal {O}_i}. \end{aligned}$$

In what follows, we will always require the time *T* to be the same for all orbits in Γ.

Next, let $L_0$ and *L* be two closed subsets (e.g., closed submanifolds) of *M*. Pick an open neighborhood U⊃L. A *U-approximate* *T-chord* from $L_0$ to *L* is an integral curve $\mathcal {O}_T(x)$ connecting $x\in L_0$ to $\varphi ^T(x)\in U$. We say that μ is a *U-approximate chord measure* associated with $L_0$ and *L* if there exists a sequence of times $T_j\rightarrow \infty$ and collections $\Gamma _j$ of *U*-approximate $T_j$-chords such that $\delta _{\Gamma _j}\rightarrow \mu$ in the weak* topology. A pseudo-chord measure associated with $L_0$ and *L* is the limit of $U_\ell$-approximate chord measures where neighborhoods $U_\ell$ shrink to *L*.

Let now $\varphi ^t$ be the Reeb flow on the boundary M=∂W of a Liouville domain *W*. Furthermore, as in Sect. 2.1, let $L_0$ and *L* be Legendrian submanifolds of *M* admitting exact Lagrangian fillings $\hat{L}_0$ and $\hat{L}$ to *W* which we can assume to be asymptotically conical. Similarly to the Hamiltonian case, we say that *L* is *accessible* from $L_0$ if for every neighborhood *U* of *L* we have $\varphi ^{T_k}(L_0)\cap U\ne \emptyset$ for some sequence $T_k\rightarrow \infty$. This is equivalent to that the set of pseudo-chord measures is non-empty, which is the case if (but not only if) $\hbar (\varphi ,L_0,L)>0$.

#### Theorem 2.7

Assume that *L* is accessible from $L_0$. Then for every U⊃L there exists a *U*-approximate chord measure associated with $L_0$ and *L* and hence also a pseudo-chord measure μ such that

$$\begin{aligned} \hbar (\varphi ; L_0,L)\le \textrm{h}_\mu (\varphi ). \end{aligned}$$

This is a version of Theorem 2.3 and Corollary 2.4 for Reeb flows. Remark 2.5 carries over to Legendrian chord measures word-for-word. Again the accessibility requirement plays a purely formal role: without it the left-hand side is zero and the right-hand side is not defined. As in the Hamiltonian case, we do not know if Theorem 2.7 holds for genuine chord measures. The proof of Theorem 2.7 is quite similar to the proof of Theorem 2.3 and is given in Sect. 3.2.

One can define approximate periodic measures and pseudo-periodic measures for flows by changing in a similar fashion discrete time to continuous time in the definitions from Sect. 2.3. Then again we have an analogue of Theorem 2.7, similar to (2.12), for such measures, but it again follows from known results.

## Proofs and refinements

### Proof of Theorem 2.3, refinements and further comments

We will prove a slightly more precise version of Theorem 2.3. To state this result, we first need to make several general definitions. As in Sect. 2.2, let $L_0$ and $L_1$ be two closed submanifolds of *M* and $\varphi :M\rightarrow M$ be a compactly supported diffeomorphism.

Fix an arbitrary Riemannian metric on *M*. Let *U* be a fixed neighborhood of *L* and $L_k:=\varphi ^k(L_0)$. We define the *volume growth entropy* for the triple $(\varphi ; L_0, U)$ as

3.1 $$\begin{aligned} \operatorname {h}_{\operatorname {vol}}(\varphi ; L_0, U) :=\limsup _{k\rightarrow \infty } \frac{\log ^+\operatorname {vol}(L_{k}\cap U)}{k}. \end{aligned}$$

Clearly, $$\operatorname {h}_{\operatorname {vol}}(\varphi ; L_0, U)$$ is independent of the background metric. Furthermore, $$\operatorname {h}_{\operatorname {vol}}$$ is monotone increasing in *U*, i.e., $$\operatorname {h}_{\operatorname {vol}}(\varphi ; L_0, U')\le \operatorname {h}_{\operatorname {vol}}(\varphi , L_0, U)$$ when $U^{\prime }\subset U$. (As a side note, we see no reason why $$\operatorname {h}_{\operatorname {vol}}$$ would have any semi-continuity properties in *U*.) Set

3.2 $$\begin{aligned} \operatorname {h}_{\operatorname {vol}}(\varphi ; L_0, L) :=\inf _{U\supset L}\operatorname {h}_{\operatorname {vol}}(\varphi ; L_0, U). \end{aligned}$$

Note that here the infimum can be replaced by the lower limit.

This definition readily translates to the framework of approximate periodic orbits in Sect. 2.3 via the graph construction. Even though we are only tangentially interested in this case, let us briefly spell it out. Let *U* be a neighborhood of the diagonal Δ in M×M and $G_k$ be the graph of $\varphi ^k$. We set

$$\begin{aligned} \operatorname {h}_{\operatorname {vol}}(\varphi ; U):=\limsup _{k\rightarrow \infty }\frac{\log ^+\operatorname {vol}(G_{k}\cap U)}{k} \text { and } \operatorname {h}_{\operatorname {vol}}(\varphi ):=\inf _{U\supset \Delta }\operatorname {h}_{\operatorname {vol}}(\varphi ; U). \end{aligned}$$

Clearly, $\operatorname {h}_{\operatorname {vol}}(\varphi ; U)$ and $\operatorname {h}_{\operatorname {vol}}(\varphi )$ are independent of the background metric, and $\operatorname {h}_{\operatorname {vol}}(\varphi ; U)$ is monotone in *U*. The infimum can be replaced by the lower limit.

#### Theorem 3.1

In the setting of Theorem 2.3, there exists a *U*-approximate chord measure μ associated with $L_0$ and *L* such that

3.3 $$\begin{aligned} \hbar (\varphi ; L_0,L)\le \operatorname {h}_{\operatorname {vol}}(\varphi ; L_0,U)\le \textrm{h}_\mu (\varphi ) \end{aligned}$$

and, as a consequence, a pseudo-chord measure μ, again associated with $L_0$ and *L*, with

$$\begin{aligned} \hbar (\varphi ; L_0,L)\le \operatorname {h}_{\operatorname {vol}}(\varphi ; L_0,L)\le \textrm{h}_\mu (\varphi ). \end{aligned}$$

Theorem 2.3 and hence Corollary 2.4 immediately follow from this result by skipping the middle terms in the inequalities. An analogue of Theorem 3.1 holds for a pseudo-periodic measure μ, i.e., $\hbar (\varphi )\le \operatorname {h}_{\operatorname {vol}}(\varphi )\le \textrm{h}_\mu (\varphi )$. However, as we have already pointed out, the first inequality is proved in [10] and the second one does not carry new information.

#### Proof of Theorem 3.1

Throughout the proof we can assume that $\hbar (\varphi , L_0,L)>0$; for otherwise the statement is void. The argument comprises two steps establishing, respectively, the first and the second inequality in (3.3).

*Step 1: From barcode entropy to volume growth.* Let $L_0$ and *L* be Hamiltonian isotopic, closed monotone Lagrangian submanifolds and φ be a Hamiltonian diffeomorphism as in the statement of the theorem. Then, by [10, Sec 5.2.3 and Cor. 5.7],

3.4 $$\begin{aligned} \hbar (\varphi ; L_0, L)\le \operatorname {h}_{\operatorname {vol}}(\varphi ; L_0, U) \end{aligned}$$

for every open set U⊃L. This proves the first part of (3.3).

*Step 2: From volume growth to separated chords to metric entropy.* Our next goal is to prove the second inequality in (3.3). This step of the proof is completely general, and it is sufficient to assume that $L_0$ and $L_1$ are two closed submanifolds of *M* and $\varphi :M\rightarrow M$ is a compactly supported $C^\infty$-smooth diffeomorphism. The $C^\infty$-smoothness requirement is essential.

#### Proposition 3.2

There exists a *U*-approximate chord measure μ associated with $L_0$ and *L* such that

$$\begin{aligned} \operatorname {h}_{\operatorname {vol}}(\varphi ; L_0, U)\le \textrm{h}_\mu (\varphi ). \end{aligned}$$

As a consequence,

$$\begin{aligned} \operatorname {h}_{\operatorname {vol}}(\varphi ; L_0, L)\le \textrm{h}_\mu (\varphi ) \end{aligned}$$

for some pseudo-chord measure μ associated with $L_0$ and *L*.

Theorem 3.1 immediately follows from (3.4), establishing the first inequality in (3.3), and Proposition 3.2 proving the second.

#### Proof

Set

$$\begin{aligned} d_k(x,y):=\max \{ d\big (\varphi ^i(x), \varphi ^i(x)\big )\mid 0 \le i\le k\}. \end{aligned}$$

We say that two *k*-orbits

$$\begin{aligned} \mathcal {O}_k(x):=\{x,\varphi (x),\ldots ,\varphi ^k(x)\} \text { and } \mathcal {O}_k(y):=\{y,\varphi (y),\ldots ,\varphi ^k(y)\} \end{aligned}$$

are η-$d_k$-separated if $d_k(x,y)>\eta$ or, in other words, $d\big (\varphi ^i(x), \varphi ^i(y)\big )>\eta$ for some $0\le i\le k$. Let $\mathcal {S}_k(\eta )$ be a maximal collection of η-$d_k$-separated points $x\in L_0$ with $\varphi ^k(x)\in U$. This set is naturally in one-to-one correspondence with the set of η-$d_k$-separated chords from $L_0$ to *U* where we identify an approximate chord with its initial condition. Note that while this collection might not be unique, the cardinality $|\mathcal {S}_k(\eta )|$ is well defined. The key to the proof is the following lemma, which is ultimately a consequence of the “local Yomdin theorem”; see [43, Thm. 1.8].

#### Lemma 3.3

Pick any constant $0<a<\operatorname {h}_{\operatorname {vol}}(\varphi ,L_0,U)$. Then, when η>0 is sufficiently small,

$$\begin{aligned} a \le \limsup _{k\rightarrow \infty }\frac{\log ^+|\mathcal {S}_{k}(\eta )|}{k}. \end{aligned}$$

#### Proof

Denote by $B_k(x,\eta )$ the $d_k$-ball of radius η centered at *x*. Choose a maximal collection $S_k(\eta )$ and fix $\eta '>\eta$, e.g., $\eta '=2\eta$. Then

$$\begin{aligned} L_k\cap U\subset \bigcup _{x\in \mathcal {S}_k(\eta )} \varphi ^k\big (B_k(x,\eta ')\big ). \end{aligned}$$

Thus

3.5 $$\begin{aligned} \operatorname {vol}(L_k\cap U)\le |\mathcal {S}_k(\eta )|\cdot \max _{x\in \mathcal {S}_k(\eta )}\operatorname {vol}\big (\varphi ^k(L_0\cap B_k(x,\eta '))\big ). \end{aligned}$$

Set

$$\begin{aligned} E_k(x,\eta '):=\frac{\log ^+ \operatorname {vol}\big (\varphi ^k(L_0\cap B_k(x,\eta '))\big )}{k}. \end{aligned}$$

Then, by (3.5),

$$\begin{aligned} \frac{\log ^+ \operatorname {vol}(L_k\cap U)}{k}\le \frac{\log ^+ |\mathcal {S}_k(\eta )|}{k}+ \max _{x\in \mathcal {S}_k(\eta )} E_k(x,\eta '). \end{aligned}$$

By [43, Thm. 1.8],

3.6 $$\begin{aligned} \lim _{\eta '\rightarrow 0+}\limsup _{k\rightarrow \infty }\sup _{x\in L_0} E_k(x,\eta ')=0 \end{aligned}$$

whenever φ is $C^\infty$-smooth. The lemma follows from (3.6). □

In the setting of Lemma 3.3, pick a sequence $k_j\rightarrow \infty$ such that

3.7 $$\begin{aligned} a \le \lim _{k_j\rightarrow \infty }\frac{\log ^+|\mathcal {S}_{k_j}(\eta )|}{k_j}. \end{aligned}$$

Let $\Gamma _{k_j}$ be the set of *U*-approximate $k_j$-chords with initial conditions $\mathcal {S}_{k_j}(\eta )$. After passing to a subsequence, we can assume that $\delta _{k_j}\rightarrow \mu$ (in the weak* topology) for some *U*-approximate chord measure μ. Clearly, $|\Gamma _{k_j}|=|\mathcal {S}_{k_j}(\eta )|$. Since $\mathcal {S}_k(\eta )$ is an η-$d_k$-separated set, by (3.7) and [25, Lemma 4.5.2],

$$\begin{aligned} a\le \lim _{k_j\rightarrow \infty } \frac{\log ^+ |\mathcal {S}_{k_j}(\eta )|}{k_j}\le \textrm{h}_\mu (\varphi ). \end{aligned}$$

Finally, by sending $a\rightarrow \operatorname {h}_{\operatorname {vol}}(\varphi ; L_0,U)$ and passing to the limit again, we obtain a *U*-approximate chord measure μ such that $\operatorname {h}_{\operatorname {vol}}(\varphi ; L_0,U)\le \textrm{h}_\mu (\varphi )$.

□

This completes the proof of Theorem 3.1. □

In the rest of this section, we will elaborate on the notions of an approximate periodic measure and a pseudo-periodic measure from Sect. 2.3. Below we make no background symplectic topological assumptions on symplectic or Lagrangian manifolds. For instance, in Proposition 3.4 we do not claim that every symplectic manifold admits a closed monotone Lagrangian submanifold.

#### Proposition 3.4

For any diffeomorphism of a closed manifold or a flow every ergodic measure is pseudo-periodic. Likewise, every ergodic measure is a Lagrangian or Legendrian pseudo-chord measure for Hamiltonian diffeomorphisms or Reeb flows for a suitable choice of Lagrangian or Legendrian submanifolds.

#### Proof

We will focus on the case of diffeomorphisms; for flows the argument is similar. Let μ be an ergodic measure. By Poincaré’s Recurrence Theorem, μ-a.e. point x∈M is recurrent, i.e., for μ-a.a. x∈M the trajectory $\{\varphi ^k(x)\mid k\in {\mathbb N}\}$ gets arbitrarily close to *x*. Furthermore, for μ-a.a. x∈M we have

$$\begin{aligned} \mu =\lim _{k\rightarrow \infty }\frac{1}{k+1}\sum _{i=0}^k \delta _{\varphi ^i(x)} \end{aligned}$$

due to Birkhoff’s Ergodic Theorem. Taking *x* in the intersection of these two full measure sets, we see that μ is an η-approximate periodic measure for any η>0. As a consequence, μ is a pseudo-periodic measure.

For chord measures it now suffices to take any Lagrangian (Legendrian) submanifolds $L_0$ and *L* intersecting at *x*. □

Revisiting Remark 2.6, recall that the limit of measures $\delta _\Gamma$ is a *periodic measure* when all orbits comprising Γ are periodic, but possibly have different periods. Such measures are automatically pseudo-periodic as the following simple observation shows.

#### Proposition 3.5

For any diffeomorphism of a closed manifold or a flow every periodic measure is pseudo-periodic.

#### Proof

Let μ be a periodic measure. We start with the discrete case. By definition, there exists a sequence $\Gamma _j$ such that $\delta _{\Gamma _j}\rightarrow \mu$ and each $\Gamma _j$ is a finite collection of periodic orbits $\mathcal {O}_{k_{ij}}(x_{ij})$. Let $k_j$ be the least common multiple of all periods $k_{ij}$ and $\Gamma '_j$ comprise the periodic orbits $\mathcal {O}_{k_j}(x_{ij})$ of the same period $k_j$. Then $\Gamma '_j$ is a collection of η-approximate periodic orbits in the sense of Sect. 2.3 for any η>0, and $\delta _{\Gamma '_j}=\delta _{\Gamma _j}$. Hence, $\mu =\lim \delta _{\Gamma '_j}$ is a genuine periodic measure in the sense of Remark 2.6 and, in particular, a pseudo-periodic measure.

For continuous time, the argument is slightly different with the exact equality of measures and periods replaced by approximations. Now we have $\delta _{\Gamma _j}\rightarrow \mu$ where each $\Gamma _j$ is a finite collection of periodic orbits $\mathcal {O}_{T_{ij}}(x_{ij})$. For every ϵ>0, there exists an arbitrarily large $T_j$ such that $|T_j-k_{ij}T_{ij}|<\epsilon$ for some integers $k_{ij}>0$. Let $\Gamma '_j$ be the multiset of the orbits $\mathcal {O}_{T_{j}}(x_{ij})$. Note that we can make the norm of the difference $\delta _{\Gamma _j}-\delta _{\Gamma '_j}$ in the dual of $C^0(M)$ arbitrarily small by making ϵ>0 small. Likewise, given η>0, the orbits $\mathcal {O}_{T_{j}}(x_{ij})$ are η-approximately periodic when ϵ is small. Thus for any sequence $\eta _\ell \rightarrow 0+$ we can find a sequence $\Gamma '_\ell$ of collections of $\eta _\ell$-approximately periodic orbits such that we still have $\mu =\lim \delta _{\Gamma '_\ell }$. □

### On the proof of Theorem 2.7

For continuous time too, it is convenient to prove a slightly more precise version of the theorem by inserting the volume growth entropy as an intermediate term. The argument follows the same line of reasoning as the proof in the previous section of Theorem 2.3 and, moreover, the theorem can be obtained as a consequence of that proof.

Let us start with preliminary definitions. In the setting of Sect. 2.4, fix a neighborhood *U* of *L* and set

$$\begin{aligned} V(s, U):=\operatorname {vol}(L_s\cap U), \end{aligned}$$

where $L_s:=\varphi ^s(L_0)$. We define the volume growth entropy of a flow φ by

3.8 $$\begin{aligned} \operatorname {h}_{\operatorname {vol}}(\varphi ; L_0, U) :=\limsup _{s\rightarrow \infty }\frac{\log ^+ V(s,U)}{s} \end{aligned}$$

and

3.9 $$\begin{aligned} \operatorname {h}_{\operatorname {vol}}(\varphi ; L_0,L) :=\lim _{U \rightarrow L}\operatorname {h}_{\operatorname {vol}}(\varphi ; L_0, U) =\inf _{U\supset L} \operatorname {h}_{\operatorname {vol}}(\varphi ; L_0, U). \end{aligned}$$

Our next goal is to connect this definition with the definition of the volume growth entropy for diffeomorphisms from the previous section. To this end, we first note that while up to this point the difference between maps and flows has always been clear from the context, it is not necessarily so below. Hence, in what follows φ refers to the flow while its time-*T* map will be denoted by $\varphi ^T$.

It is easy to see that for any T>0 and any U⊃L, we have

3.10 $$\begin{aligned} T^{-1}\operatorname {h}_{\operatorname {vol}}(\varphi ^T;L_0, U)\le \operatorname {h}_{\operatorname {vol}}(\varphi ;L_0, U). \end{aligned}$$

Here the volume growth entropy of the flow φ is understood as in (3.8) and (3.9), while the volume growth entropy of the time-*T* map $\varphi ^T$ is defined in (3.1) and (3.2). The opposite inequality, given by the following lemma, is more subtle.

#### Lemma 3.6

Assume that the closure of an open set $U^{\prime }⊃L$ is contained in *U*. Then, whenever T>0 is sufficiently small (depending on $U^{\prime }$ and *U*), we have

3.11 $$\begin{aligned} \operatorname {h}_{\operatorname {vol}}(\varphi ; L_0, U')\le T^{-1}\operatorname {h}_{\operatorname {vol}}(\varphi ^T; L_0, U). \end{aligned}$$

#### Remark 3.7

In Lemma 3.6, the smaller the gap between $U^{\prime }$ and *U*, the smaller the required value of *T* is. We do not know if *T* can be chosen independent of the gap and if we can have $$\operatorname {h}_{\operatorname {vol}}(\varphi ; L_0, U)= T^{-1}\operatorname {h}_{\operatorname {vol}}(\varphi ^T; L_0, U)$$ and $$\operatorname {h}_{\operatorname {vol}}(\varphi ; L_0, L)= T^{-1}\operatorname {h}_{\operatorname {vol}}(\varphi ^T; L_0, L)$$ for some specific value of *T*. The underlying reason is the lack of any clear-cut continuity of $$\operatorname {h}_{\operatorname {vol}}$$ with respect to *U*.

#### Proof

Let T>0 be so small that $\varphi ^\tau (U')\subset U$ whenever $|\tau |\le T$. Pick a sequence $s_j\rightarrow \infty$ such that

$$\begin{aligned} \lim _{j\rightarrow \infty }\frac{\log ^+ V(s_j,U')}{s_j}=\operatorname {h}_{\operatorname {vol}}(\varphi ;U'). \end{aligned}$$

Then for some $k_j\in {\mathbb N}$, we have $s_j=k_j T+\tau _j$ where $|\tau _j|\le T$. Hence,

$$\begin{aligned} V(s_j,U')\le C\cdot V(k_jT,U) \end{aligned}$$

for some constant C>0 independent of $s_j$, and (3.11) follows. □

Let now M=∂W be the boundary of a Liouville domain *W* and $\varphi ^t$ the Reeb flow on *M*. Furthermore, as in Sect. 2.1, let $L_0$ and *L* be closed Legendrian submanifolds with exact compact Lagrangian fillings to *W*. Fix an open set U⊃L in *M*.

#### Theorem 3.8

In the setting of Theorem 2.7, for every neighborhood $U^{\prime }⊃L$ whose closure is contained in *U*, there exists a *U*-approximate chord measure μ associated with $$L_0$$ and *L* such that

3.12 $$\begin{aligned} \hbar (\varphi ; L_0,L)\le \operatorname {h}_{\operatorname {vol}}(\varphi ;L_0,U')\le \textrm{h}_\mu (\varphi ). \end{aligned}$$

Hence, there is also a pseudo-chord measure μ, again associated with $L_0$ and *L*, with

$$\begin{aligned} \hbar (\varphi ; L_0,L)\le \operatorname {h}_{\operatorname {vol}}(\varphi ;L_0,L)\le \textrm{h}_\mu (\varphi ). \end{aligned}$$

#### Proof

The first inequality in (3.12) is an analogue of (3.4) for Reeb flows; it is established in [17]. Hence we only need to prove the second inequality.

Assume now that the closure of a neighborhood $U^{\prime }⊃L$ is contained in *U*. Then, by Lemma 3.6,

$$\begin{aligned} \operatorname {h}_{\operatorname {vol}}(\varphi ; L_0, U')\le T^{-1}\operatorname {h}_{\operatorname {vol}}(\varphi ^T; L_0, U) \end{aligned}$$

for all sufficiently small T>0. By Proposition 3.2 applied to $\varphi ^T$ we have

$$\begin{aligned} \operatorname {h}_{\operatorname {vol}}(\varphi ^T; L_0, U)\le \textrm{h}_\mu (\varphi ^T) \end{aligned}$$

for some *U*-approximate $\varphi ^T$-chord measure μ. It is easy to see that the measure μ is automatically invariant under the flow φ and also, by construction, a *U*-approximate chord measure for the flow. Furthermore,

$$\begin{aligned} \textrm{h}_\mu (\varphi ^T)=T\textrm{h}_\mu (\varphi ^1)=T\textrm{h}_\mu (\varphi ). \end{aligned}$$

Combining the last two displayed formulas, we obtain the second inequality in (3.12). □

Likewise, in the case of approximate periodic orbits, we fix a neighborhood *U* of the diagonal Δ in M×M. For instance, we can take $U=U_\eta$ to be the η-neighborhood of Δ as in (2.11). Denote by $G_s$ the graph of $\varphi ^s$ in M×M and set

$$\begin{aligned} V(s,U):=\operatorname {vol}(G_s\cap U_\eta ) \end{aligned}$$

Then the volume growth entropy, $\operatorname {h}_{\operatorname {vol}}(\varphi ; U)$ and $\operatorname {h}_{\operatorname {vol}}(\varphi )$, of the flow φ is still defined by (3.8) and (3.9). The upper bound (3.11) from Lemma 3.6 takes the form $\operatorname {h}_{\operatorname {vol}}(\varphi ; U')\le T^{-1}\operatorname {h}_{\operatorname {vol}}(\varphi ^T; U)$. Again we have an analogue of Theorem 3.8, but it does not contain new information as stated because every ergodic measure is pseudo-periodic. The first inequality in this version of the theorem follows from the main result of [16] (see also [14]) and the second one from the existence of maximal entropy ergodic measures; [35].

## Examples

In this section, we discuss several examples further illustrating the notion of a pseudo-chord measure.

### Example 4.1

(Hyperbolic sets). Let *K* be a locally maximal, topologically transitive compact hyperbolic set of a Hamiltonian diffeomorphism $\varphi :M\rightarrow M$. Assume that $L_0$ and *L* intersect *K* and that $L_0$ contains a ball centered at $p\in L_0\cap K$ in the unstable manifold of *K* and *L* contains a ball centered at $q\in L\cap K$ in the stable manifold. Then, as shown in [34], $\operatorname {h}_{\operatorname {top}}(\varphi |_K)\le \hbar (\varphi ;L_0,L)$. Thus, we have

4.1 $$\begin{aligned} \operatorname {h}_{\operatorname {top}}(\varphi |_K)\le \hbar (\varphi ;L_0,L)\le \textrm{h}_\mu (\varphi )\le \operatorname {h}_{\operatorname {top}}(\varphi ), \end{aligned}$$

where all inequalities can be strict. A similar chain of inequalities holds for Legendrian submanifolds and hyperbolic sets of Reeb flows with the first inequality established in [18].

The next two examples concern with the cases where φ is globally hyperbolic and the pseudo-chord measure can be explicitly identified as the maximal entropy measure.

### Example 4.2

(Hyperbolic symplectomorphisms) Let $\varphi :{\mathbb T}^{2}\rightarrow {\mathbb T}^{2}$ be a hyperbolic symplectomorphism. For instance, φ itself can be a hyperbolic linear automorphism. Furthermore, let $L_0$ and *L* be two embedded contractible circles in ${\mathbb T}^2$ bounding the same area. Thus $L_0$ and *L* are Hamiltonian isotopic monotone Lagrangian submanifolds of ${\mathbb T}^{2}$. Then $L_k:=\varphi ^k(L_0)$ is still Hamiltonian isotopic to *L*. Hence, the filtered Floer homology of the pair $(L,L_k)$, and hence the barcode, are defined. The results and constructions from [10, 34] and Sect. 2.2 carry over to this case.

Assume, in addition, that $L_0$ contains an arc in an unstable leaf of φ and *L* contains an arc in a stable leaf. Note that while stable/unstable foliations are only $C^0$-smooth, individual leafs are $C^\infty$. Then the results of [34] still apply with $K={\mathbb T}^{2}$, and the inequalities in (4.1) turn into the equality

4.2 $$\begin{aligned} \hbar (\varphi ;L_0,L)=\textrm{h}_\mu (\varphi )=\operatorname {h}_{\operatorname {top}}(\varphi ). \end{aligned}$$

Hence, in this case, the pseudo-chord measure μ is the unique maximal entropy measure, the Bowen measure [25, Sec. 20.1]. If φ is linear, μ is the normalized Lebesgue measure. We do not know what the measure μ is when the stable/unstable leaf condition is dropped. (Note however that $L_0$ and *L* are automatically tangent to the unstable and stable foliations.)

We expect this example to generalize to hyperbolic symplectomorphisms $\varphi :{\mathbb T}^{2n}\rightarrow {\mathbb T}^{2n}$ and Hamiltonian isotopic monotone Lagrangian submanifolds $L_0$ and *L* of ${\mathbb T}^{2n}$. However, it is not clear how to guarantee that then $L_k$ and *L* are still Hamiltonian isotopic even when φ Hamiltonian isotopic to a linear automorphism. Furthermore, when 2n≥4, the tangency condition is not generically satisfied for dimension reasons.

### Example 4.3

(Geodesic flows without conjugate points) Let *Q* be a closed manifold equipped with a metric *g* without conjugate points and φ be the geodesic flow on the unit (co)tangent bundle $M:=ST^{*}Q$. For instance, *g* can be a metric of negative sectional curvature and then φ is a hyperbolic (aka Anosov) Reeb flow.

As $L_0$ and *L* we take the unit spheres $ST^{*}_pM$ and $ST^{*}_qM$ for two not necessarily distinct points *p* and *q* in *M*. These Legendrian submanifolds are nowhere tangent to the stable/unstable foliations of φ. As a consequence, even when φ is hyperbolic, $L_0$ and $L_1$ do not meet the stable/unstable manifold condition from [18] alluded to in Example 4.1 for the hyperbolic set *M*. Thus, we cannot use that criterion to show that

$$\begin{aligned} \hbar (\varphi ;L_0,L)=\operatorname {h}_{\operatorname {top}}(\varphi ). \end{aligned}$$

However, this equality still holds.

To see this, recall that the wrapped Floer homology persistence module for the pair $(L_0,L)$ is isomorphic to the Morse homology persistence module for the space of paths connecting *p* and *q* filtered by length [2]. (In fact, in this setting we could have directly used the Morse-theoretic version of barcode entropy from [20].) The latter Morse complex does not have finite bars and ${\mathfrak b}_\epsilon (s;L_0,L)={\mathfrak b}_\infty (s;L_0,L)$ is simply the number of geodesics of length below *s* connecting *p* and *q*. By [32], the exponential growth rate of this number as a function of *s* is equal to $\operatorname {h}_{\operatorname {top}}(\varphi )$. (The condition that *g* has no conjugate points is sufficient here.)

Therefore, the analogue of (4.2) holds in this case for some pseudo-chord measure μ, which is then a measure of maximal entropy. The measure is unique when φ is hyperbolic, and this is the Bowen–Margulis measure [25, Sec. 20.5]. These observations also carry over to the case of pseudo-periodic measures, but then the result is entirely expected.

## Data Availability

No datasets were generated or analysed during the current study.
